# Defining the noma research agenda

**DOI:** 10.1371/journal.pntd.0012940

**Published:** 2025-04-21

**Authors:** Anaïs Galli, Marianne Comparet, Daniel Argaw Dagne, Denise Baratti-Mayer, Thi H. Cao, Philippe J. Guérin, Maria Guevara, Manuel W. Hetzel, Claire Jeantet, Emmanuel Kabengele Mpinga, Valter Muendane, Mulikat Okanlawon, Erika Placella, Marta Ribes, Mark Sherlock, Jürg Utzinger, Peter Steinmann

**Affiliations:** 1 Swiss Tropical and Public Health Institute, Allschwil, Switzerland; 2 University of Basel, Basel, Switzerland; 3 International Society for Neglected Tropical Diseases, London, United Kingdom; 4 World Health Organization, Geneva, Switzerland; 5 Hôpitaux Universitaires de Genève, Geneva, Switzerland; 6 Drugs for Neglected Diseases initiative (DNDi), Geneva, Switzerland; 7 Infectious Diseases Data Observatory (IDDO), University of Oxford, Oxford, United Kingdom; 8 Centre for Tropical Medicine and Global Health, Nuffield Department of Medicine, University of Oxford, Oxford, United Kingdom; 9 Médecins Sans Frontières, Geneva, Switzerland; 10 Inediz, Saint-Cyr-sur-Mer, France; 11 Elysium Noma Survivors Association, Basel, Switzerland; 12 Institute of Global Health, University of Geneva, Geneva, Switzerland; 13 Swiss Agency for Development and Cooperation, Bern, Switzerland; 14 Barcelona Institute for Global Health, Barcelona, Spain; 15 Faculty of Medicine and Health Sciences, University of Barcelona (UB), Barcelona, Spain; 16 Médecins Sans Frontières, Amsterdam, Netherlands; Yale University School of Medicine, UNITED STATES OF AMERICA

## Abstract

• A 1-day symposium brought together over 100 individuals with lived experience of noma, expertise in neglected tropical diseases, and public health, including researchers, health advocates, and clinicians. The involvement of noma survivors was invaluable and added an important perspective in defining the research agenda.

• The most pressing research needs identified were:

○ Clear case definition of noma

○ Early case detection and robust surveillance

○ Psychosocial and economic impact of noma

○ Decision support for diagnosing acute necrotizing gingivitis and associated antibiotic regimen(s) with treatment duration

○ Deeper understanding of risk factors and social determinants

○ Identification of effective information, education, and communication strategies

○ Effectiveness of surgical services

○ Testing decentralized follow-up for patients

• An important conclusion was that noma research and control activities must be integrated across sectors and disciplines, such as neglected tropical diseases, oral health, nutrition, and child health programs including immunization.

## Introduction

**Fig 1:**
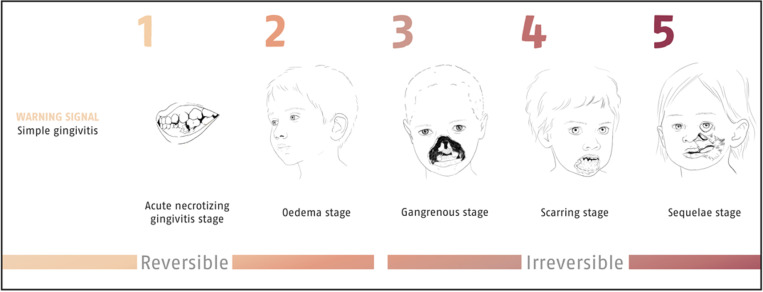
The five stages of noma (1–5), characterized in four localization types [ 4,5]. Figure drawn by Chloé Fournier/Inediz.

Noma is a severely debilitating oro-facial gangrenous disease, mostly affecting children aged 2–6 years [1]. Thus far, no specific causative agent of noma has been identified [2]. Yet, a large variety of risk factors linked to poverty and inequity have been reported, such as chronic malnutrition, having previously suffered from an infectious disease, poor oral hygiene, and no or very limited access to water, sanitation, and hygiene (WASH) infrastructure [3]. Noma may develop if an individual’s immune system is weakened, the oral microbiome is altered, and bacteria commonly present in the mouth shift to a pathogenic profile. Noma can be categorized into five stages: 1, acute necrotizing gingivitis (ANG); 2, edema; 3, gangrene; 4, scarring; and 5, sequelae [4] (Fig 1). Up to stage 2, noma is reversible with timely broad-spectrum antibiotic treatment, nutritional support, and oral hygiene. From stage 3 onward, functional impairment and facial disfigurement become permanent. According to the gravity of the sequela, noma survivors require access to reconstructive surgery to facilitate a functional restoration allowing them to drink, eat, speak, and breathe more easily and to support re-integration into society by reducing stigmatization linked to the disfigurement [1]. However, access to reconstructive surgery remains challenging, either not available or not affordable, in most settings where noma occurs.

**Fig 2:**
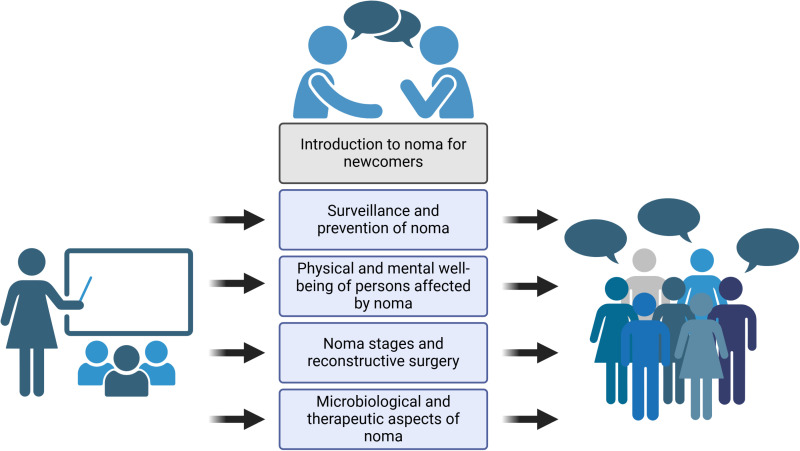
Structure and content covered in the 1-day symposium *Defining the Noma Research Agenda*, held on 20 September 2024 at Swiss TPH in Allschwil, Switzerland. Created with Biorender.

Current epidemiologic data about the global incidence, mortality, and geographic distribution of noma is scarce [6]. Due to the rapid progression of the disease, which often results in undocumented deaths outside of health facilities, stigmatization in the community, limited access to health care, and poor identification of noma by health workers, noma is not accurately reported. The mortality of untreated cases is believed to be very high, though evidence is scarce to ascertain figures [2]. Among patients receiving timely antibiotic treatment, mortality has been reported to range between 0% and 38% (including patients with immunodeficiency) [3]. The most widely cited global incidence estimate of noma stems from a World Health Organization (WHO) Delphi consultation dating back to 1998, suggesting 140,000 new cases per year [7]. The same consultation put forth a global prevalence of 770,000 people living with sequelae of the disease. Newer studies reporting noma prevalence or incidence vary greatly in their conclusions and are limited by poor study designs. The latest estimate on global incidence published in 2003 reported 30,000-40,000 noma cases annually [8]. Contrary to common belief, noma is not confined to the African continent, but occurs in most low- and middle-income countries and sporadic cases continue to occur worldwide [2,6].

Interest in noma has increased since December 2023 when WHO officially declared it a neglected tropical disease (NTD) [9]. Additionally, two noma survivors, Mulikat Okanlawon (co-author) and Fidel Strub, were recognized internationally as two strong health advocates of the impact of noma by the TIME 100 Health 2024 list [10]. In order to build on this unprecedented momentum, the Swiss Tropical and Public Health Institute (Swiss TPH) and Elysium, Noma Survivors’ Association, joined forces and convened a 1-day symposium. The event brought together more than 100 people with lived experience of the disease, donors, noma and NTDs experts, and other public health specialists, including researchers, health advocates, and clinicians with first-hand experience in the treatment and management of noma. The aim of the symposium was two-fold. First, to review the current understanding of noma and to define a research agenda to generate new evidence and shape a strategy to prevent, control, and eliminate noma as a public health problem. Second, to reflect on how the identified research priorities fit into a broader framework of NTDs, highlighting potential synergies in advocacy, research and development (R&D), prevention, control, and elimination of noma and other NTDs.

## Methods

The symposium *“Defining the Noma Research Agenda”* took place on 20 September 2024 at Swiss TPH in Allschwil, Switzerland, and was open to interested individuals and organizations in a hybrid format for plenary sessions (Fig 2). During the morning, experts introduced noma to the audience, including the latest clinical and epidemiologic research, while a panel discussion with noma survivors set the stage for sharing the realities of surviving noma and living with its life-long consequences. In the afternoon, participants split into four thematic breakout groups, accompanied by a note taker and rapporteur to (i) brainstorm; (ii) condense the discussion into specific research questions; and (iii) develop concepts for research projects or programs (e.g., setting, size, funding, and partners). The focal areas of the breakout groups were:

- surveillance and prevention of noma
- physical and mental well-being of persons affected by noma
- noma stages and reconstructive surgery
- microbiologic and therapeutic aspects of noma

A fifth breakout group was open to participants who were new to the topic and interested in receiving an in-depth introduction on current knowledge of noma. Once reunited in the plenary, the rapporteurs summarized the discussion outcomes, followed by reactions in the plenary, identification of cross-cutting themes, discussion on overall priorities, and exploring potential synergies to the broader group of NTDs.

## Results

### Symposium participants

**Fig 3:**
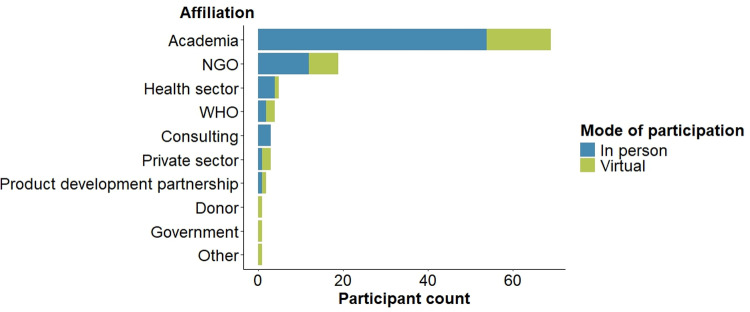
Characteristics of noma symposium participants who have participated in-person (blue) or for online attendance (green).

Participants at the 1-day symposium were affiliated with academia, non-governmental organizations (NGOs), public health institutions, government and private sector, donor community, WHO, and product development partnerships (Fig 3). Overall, 77 people participated physically and 30 were present online, including speakers and moderators.

### Identified research needs

During the four thematic sessions, the following topics were identified as most pressing:

1. *Clear case definition:* A commonly applied standard protocol based on the WHO-defined noma stages is needed to improve the accuracy and validity of data, facilitate early detection, and ensure the quality of diagnosis and care. Standardized reporting is even more pressing as a foundation for research and surveillance. Of note, with most noma cases occurring in remote rural areas away from formal health infrastructures, clear and pragmatic definitions are instrumental in supporting all actors formally or informally involved in primary healthcare at the community level, including traditional healers.
2. *Early case detection and robust surveillance:* Health information and reporting systems adapted to remote and resource-constrained settings are critical for capturing data on noma cases and affected populations, thus determining the noma disease distribution and burden as accurately as possible. In addition, detecting cases early and collecting essential data on demographic characteristics, the occurrence and clinical presentation of noma patients will provide the evidence base for timely targeted prevention and response focusing on areas and populations most at risk. Digital technology, such as mapping of high-risk areas, and integration with other surveillance platforms and preventive programs, including for other NTDs or childhood conditions, offers opportunities for synergistic management and control. A robust case detection and data collection framework is essential in ensuring the protection of highly sensitive data, collected in some of the most neglected and vulnerable areas and populations.
3. *Psychosocial and economic impact of noma:* People affected by noma and their families are often faced with significant barriers complicating their reintegration into society. These barriers include stigmatization, traditional beliefs, lack of schooling, difficulties finding a job, and living with physical and mental trauma. Participatory research is needed to determine not only the barriers but also amplify the direct input, views, and voices of affected persons and their communities regarding the best interventions to overcome these challenges. Evidence is required on the psychosocial and economic burden and mental health benefits of initiatives aiming to reintegrate noma survivors into society, such as mentorship programs, community initiatives, and support groups.
4. *Decision support for diagnosing ANG and associated antibiotic regimen(s) with treatment duration:* Evidence-based guidance is required on who is at risk of progressing from ANG to noma and which antibiotic drug combinations are recommended at what doses and length of time, including possible regional differences [4]. This will promote prompt and effective treatment and follow-up, prevention of severe cases, and mitigate the risk of the development and spread of antimicrobial resistance.
5. *Risk factors and social determinants:* Research to better characterize risk factors, social determinants, and inequities will aid in the understanding of the pathogenesis of noma and inform targeted prevention, surveillance, and early detection. The following areas have been identified as particularly relevant for in-depth studies: genetic factors, prior exposure to infectious diseases, immunologic signatures, behavior, WASH conditions, parity of mother, demographic factors (e.g., strong clustering in young age group, no family clustering, sex, and gender), and regional variation (e.g., stage duration).
6. *Identification of effective information, education, and communication (IEC) strategies:* Raising awareness and promoting health among the public as well as health personnel is challenging due to the general neglect of noma, the widespread ignorance regarding the condition and the stigma associated with the disease. Consequently, the condition may be referred to with particular terms that vary between socio-cultural groups, challenging IEC in endemic settings that are multi-lingual, multi-ethnic, or multi-cultural. One priority in this field would be identifying communication strategies specifically targeted to populations at risk. Additionally, there is a need for sensitization on the early stages of the disease and measures to prevent it with a focus on pregnant women, mothers and guardians of young children, traditional healers, and health workers in peripheral facilities. To ensure awareness, prompt diagnosis and treatment, representation of noma in relevant medical, dentistry, and nursing training curricula is also warranted.
7. *Effectiveness of surgical services:* Progress has been made with establishing surgical capacity in selected endemic countries, but the high degree of specialization of staff and equipment will probably continue to require a proportion of cases to be treated either by mobile teams or in central locations. Establishing and evaluating a network of referral centers with the prerequisite capacity is thus paramount. Such centers are ideally located in endemic countries and address surgical needs beyond noma, but may also be located in high-income countries. Additionally, ways to support family members accompanying noma patients during the extended treatment and rehabilitation periods should be investigated.
8. *Testing decentralized follow-up:* Mobile digital technology may offer novel approaches to reduce the length of stay in rehabilitation centers and thus the burden on families, and improve follow-up. Digital technology may also facilitate the formation of self-help groups of persons with lived experience of noma, catch-up education, and other rehabilitation-related activities. Such tools may also facilitate the systematic collection of relevant data (see point 2).

## Discussion and way forward

Much of the challenges that were discussed in the symposium pertain to the severe neglect of noma and the affected and highly vulnerable populations. First, the five noma stages defined by WHO are only used sparsely by stakeholders in reporting, rendering comparability of noma-related data challenging [4]. Consequently, we advise emphasizing their relevance, so that a unified nomenclature is used across disciplines and communities. Second, the lack of knowledge about noma among clinicians, nurses, and other public health professionals is limiting the feasibility of broad-based consultations, early case detection, and surveillance activities. Noma diagnosis, clinical presentations, and treatment need to be integrated into health curricula and educational campaigns, with a focus on differential diagnosis and prompt interventions to reduce the risk of irreversible damage and premature death. Third, the absence of even basic epidemiologic data on noma is simply unacceptable. It is paramount to establish active and passive surveillance on a global scale and to integrate noma into (national) health information systems and campaigns that include the most marginalized and vulnerable populations. To start addressing the neglect, noma research should be pragmatic and not over-medicalized to strengthen early case detection, thus averting the disease’s progression to an irreversible stage.

A major asset of the symposium was the active participation of noma survivors. Efforts should be made to facilitate their permanent presence in research and advocacy as pioneered for other conditions. Survivors' priorities were not always aligned with what academics thought to be necessary. Discussions concluded on the importance of considering the socio-economic and mental health needs of people affected by noma and their families. Having mentors and support from a community of like-minded peers or people with similar experiences, such as the pilot program *Experts by Experience* by Médecins sans Frontières, proved extremely valuable for survivors. Hence, associations of people with lived experience of noma need to be strengthened locally and globally. A potential guide for such collaborations could be the WHO guidelines for meaningful engagement of people with lived experience [11], the fact sheet of the Swiss Clinical Trial Organisation about patient and public involvement [12], or the 11 principles of the Commission for Research Partnerships with Developing Countries (KFPE) [13].

Finally, in line with its recent addition to the WHO list of NTDs, noma should be fully integrated into the NTD research, control, and elimination agenda. The integrated skin NTDs framework, for example, provides a promising fit for noma, and elaborates on how resources for diagnosis, wound care, detection and preventive campaigns, support groups, and advocacy with people affected should be used [14]. Addressing NTDs together offers specific opportunities for funding bodies and policymakers through reduced fragmentation and improved cost structures. While basic research will be instrumental in elucidating some of the pressing knowledge gaps around noma, control and care will be boosted with a step-up in operational research, and data to improve access to health care and prevention. This focus is in line with the effort to achieve Universal Health Coverage and realize the Sustainable Development Goal 3 pertaining to health. Recognized by the Human Rights Council in 2012 as “the most brutal face of poverty and malnutrition” [15], noma truly is an indicator for inequities.
